# Regional well-being inequalities arising from healthcare expenditure public policies in Spain

**DOI:** 10.3389/fpubh.2022.953827

**Published:** 2022-09-21

**Authors:** María del Carmen Valls Martínez, Mayra Soledad Grasso, José-María Montero

**Affiliations:** ^1^European Research Center on Economics and Sustainable Development, Economics and Business Department, University of Almería, Almería, Spain; ^2^Department of Political Economy and Public Finance, Economic and Business Statistics, and Economic Policy, University of Castilla-La Mancha, Toledo, Spain

**Keywords:** Spanish health system, life expectancy, well-being inequalities, healthcare expenditure public policies, partial least squares structural equation modeling, cluster analysis, principal component analysis

## Abstract

Well-being inequalities arising from different healthcare expenditure public policies is currently a hot topic at a national scale, but especially so at a sub-national level because the inequalities in question are among citizens of the same country. Spain is an optimal study area to carry out research on this topic because it is considered to have one of the best health systems in the world, it is one of the top-ranking countries in terms of life expectancy rates (the indicators we use for well-being), and it has a decentralized public health system with significantly different regional healthcare expenditure public policies. Given that the factors involved in the complex direct, indirect, and second-order relationships between well-being and health spending are latent in nature, and that there are more hypotheses than certainties regarding these relationships, we propose a partial least squares structural equation modeling specification to test the research hypotheses and to estimate the corresponding impacts. These constructs are proxied by a set of 26 indicators, for which annual values at a regional scale were used for the period 2005–2018. From the estimation of this model, it can be concluded that mortality, expenditure and resources are the factors that have the greatest impact on well-being. In addition, a cluster analysis of the indicators for the constructs included in this research reveals the existence of three clearly differentiated groups of autonomous communities: the northern part of the country plus Extremadura (characterized by the lowest well-being and the highest mortality rates), Madrid (with the best results in well-being and mortality, the lowest public health expenditure per inhabitant and percentage of pharmaceutical spending, and the highest percentage in specialty care services and medical staff spending), and the rest of the country (south-eastern regions, with similar well-being values to those of the first group but with less health expenditure). Finally, a principal component analysis reveals that “healthiness” and “basic spending” are the optimal factors for mapping well-being and health spending in Spain.

## Introduction

A key question when approaching research on health spending is what modern societies are primarily seeking to achieve with such spending. In the earliest literature on the topic, a variety of answers can be found, including wellness, well-being, psychological well-being, flourishing, mental health, quality of life, life satisfaction, and hedonic well-being (1–6).

The selection of the optimal “response variable” is an intellectual challenge and remains a hot topic, with contributions from multiple, widely differing scientific disciplines (7–12). In this research, we initially selected the concepts of wellness and well-being as the “response variable,” because they are two overarching concepts, which incorporate practically all the individual or partial responses (dimensions) cited in the literature. However, choosing one of them is an extremely difficult task.

Wellness, as defined for the first time in Dunn (13), refers to a special state of individual health which sees man as consisting of body, spirit and mind, and which depends on his environment. Three years after the pioneering concept put forward by Dunn, the World Health Organization (WHO) defined health in terms of wellness as “physical, mental, and social well-being” (14). Therefore, wellness is a global concept, and while physical and mental health contribute to it, they are not the only contributors. In the literature on the topic, after the WHO definition of wellness, the social, occupational, spiritual, intellectual and emotional dimensions are also cited as drivers for wellness (15). Hattie et al. (16) consider the creative self, coping self, social self and the essential self as the wellness dimensions that complement the physical self-dimension. Ardell (17), Myers et al. (18), Stoewen (19) are other interesting papers contributing to this debate. There is even some literature suggesting that wellness is a concept that is much more closely linked to happiness than to health [the pioneer in this line is Saracci (20)]. However, there is a consensus in the wellness literature that the core dimensions are physical and mental (21–24).

As for well-being, as expected, there is no consensus on a simple definition of the concept. However, as stated in CDC (25) and the literature cited therein, there is general agreement on the following aspects: well-being includes the presence of positive emotions and moods (e.g., contentment, happiness), the absence of negative emotions (e.g., depression, anxiety), satisfaction with life, fulfillment, and positive functioning. Therefore, well-being can to an extent be identified with judging life positively and feeling good. Obviously, from a public health perspective, physical (and recently mental) well-being is seen as critical to overall well-being. However, this is not the only aspect of well-being. Overall well-being also includes socio-economic well-being, development and activity, emotional well-being, psychological well-being, life satisfaction, domain specific satisfaction and engaging activities and work [see CDC (25) and the references therein].

In this research, we focus on well-being rather than on wellness. There are three reasons for this decision:

- As stated on Dr. Brandt's blog (26), wellness and well-being are like siblings: related, but different. Both are intimately linked to good health, and the two go hand-in-hand but are different. Wellness is a set of habits and behaviors, while well-being is a state of mind. In other words, wellness is a practice that can lead to greater well-being. It could be said that wellness is an important element of overall well-being, and that well-being is a more inclusive concept than wellness.
- In some branches of the literature, wellness is often identified with individuals and well-being with groups of individuals.
- In post COVID pandemic times, well-being is even more essential than ever. We have been reminded how important health and well-being are from all angles, including the economic one. As noted in Gonzalez-Natal et al. (27), well-being has now become a multistakeholder, multisectoral and multidimensional concept and people's expectations change very quickly. Specifically, the multidimensional aspect of the current concept of well-being is one of the reasons for the evolution from wellness to well-being. Therefore, it is no surprise that governments are implementing programs focused on the well-being of their citizens.

Having decided that well-being is the optimal response variable, the focus moves to how to measure well-being. From the above paragraphs, it can be easily deduced that well-being is a subjective concept. Accordingly, self-evaluations are typically used to measure it at an individual level, although objective indicators such as household income, neighborhood crime rate, etc., are also frequently used (28–30). None of them yield a complete assessment of well-being because this concept is in essence multidimensional, requiring a multimethod assessment approach. According to Alexandrova (31), at least three approaches to the concept of well-being can be distinguished: hedonic balance, life satisfaction and a version of Aristotle's idea of eudaimonia. The more than 4,000 papers per year that are currently published in Web of Science indexed journals on well-being-related topics can be framed within these approaches. The literature is also abundant on current ways of well-being measurement. By way of example, Ong et al. (32) review 240 studies published between 2005 and 2019.

However, our focus is on well-being at an aggregate scale; in other words, we are interested in community well-being (specifically Spanish well-being). Community well-being is a relatively new concept in the literature on the topic and is closely related to public health. It is not the sum of individual well-beings; it has its own entity. What is more, community well-being is necessary for individual well-being [see Kim et al. (33), and the references therein for details and discussion on this topic]. Having said that, as at the individual scale, and borrowing the words of Barrington-Leigh (34), there is a key question to be answered: Can a single number or index capture society's well-being well-enough to guide all policy decisions? This is an undeniably seductive idea that points to multidimensional indexes. However, given that multidimensional indexes are not free from subjectivism or measurement errors, a second question that needs answering is: Can a single variable (not an index but only a register) meet the above goal? Most of the recent literature points to life expectancy because it rests on a number of key dimensions related to health, housing and environment conditions, food quality, and other aspects of human life (17).

The second decision to take when approaching research on the relationship between healthcare expenditure policies and well-being at a sub-national level is the country that is the focus of the research. Obviously, the ideal would be to analyze the above relationship in all countries with a decentralized health system; however, such a task goes beyond the scope of this article and constitutes a statistical challenge from the point of view of the massive database needed to feed the statistical strategies implemented to obtain the associated estimates.

In light of the above limitation, we focus our research on Spain. At a national scale, the Spanish Public Health System is considered one of the best public health systems in the world from several perspectives: completeness, efficiency, care universalization, accessibility, primary care, quality of specialized care, spatial location, and decentralization in the management of health resources, among others. As a result, in 2020 (latest data available) Spain, together with Switzerland, had the highest life expectancy at birth in Europe (83.9 years) and was sixth in the world after Hong Kong, Japan, Macau, Switzerland and Singapore (35). This position, together with the fact that Spain also has one of the lowest mortality rates from preventable and treatable causes, suggests that public health and healthcare interventions are effective in preventing premature mortality.

The second reason for focusing our research on Spain is that, in 1986, responsibility for healthcare was decentralized from the central government to the autonomous communities, which allows us to study inter-regional disparities. Consequently, despite the existence of an Inter-territorial Council of the National Health System, whose main responsibility is to ensure equal access to health services throughout the national territory (36), it could be said that in Spain there are 17 different (regional) health systems, each with its own spending policy. Some of them claim to be the best regional public health system in the country, with the best—or one of the best—public health systems in the world. As an example, the president of the region of Madrid recently claimed that the region held first position in the ranking of Spanish regional public health systems, citing the fact that the top three positions in the Hospital Excellence Index 2021 were taken by hospitals of the region of Madrid. Therefore, the following questions arise: What about the disparity in the inter-regional healthcare systems? Does it correspond to a disparity in regional public health expenditure? In other words, is there a direct relationship between public health expenditure policies and well-being? Are there differentiated clusters of areas according to well-being indicators?

The COVID pandemic could be considered a good stress test for the national (or sub-national) public health systems' effectiveness and efficiency when facing extreme situations. Spain was one of the countries most affected by the pandemic in terms of deaths per million inhabitants. It ranks 13th in the world and 5th in Europe (37). Sachs et al. (38) developed an overall index of epidemic control for 33 OECD countries (all of them except Chile, Colombia, Mexico and Iceland), by combining the data on COVID-19 mortality rates, effective reproduction rates, and epidemic control efficiency. Spain was the worst performing country, being strongly penalized for its poor efficiency in the control of the pandemic from the perspective of reductions in daily contacts. Accordingly, we do not consider pandemic-based data as an indicator for the performance of the Spanish public health system, because its performance at that time has more to do with the political management of the pandemic and with Spain being one of the world's major travel hubs than with the performance of the public (and private) health system. There is no doubt that Spain demonstrated a low level of control, not only in airports but also of transmission across the country.

The National Health System in Spain was created in 1908 and has changed over the years. Initially, it was a centralized system, that is, it depended on the Spanish national government. However, with the Spanish Constitution of 1978, which designed the “State of Autonomies,” a process of decentralization began that has resulted in a transfer of responsibilities, including responsibility for health care, to the autonomous governments (39, 40). This has been a lengthy process and has occurred unevenly among the different regions. There are different reasons for decentralizing a country's healthcare system, such as the pursuit of public efficiency. However, the real reason behind the Spanish situation is territorial identity. In fact, the process had begun and made substantial progress even before the establishment of general (health-related) criteria on the functioning of the health system and the distribution of responsibilities to the autonomous communities, which occurred with the introduction of General Health Law 14/1986.

The so-called historical autonomous communities (Cataluña, País Vasco and Galicia), as well as the assimilated autonomous communities (Andalucía, Navarra, Comunidad Valenciana and Islas Canarias) assumed responsibility for the legislative development and management of healthcare from the beginning, in the period 1980–1999. However, in the period 2000–2007, the remaining 10 autonomous communities (Aragón, Castilla-León, Islas Baleares, Castilla-La Mancha, Extremadura, Asturias, Cantabria, Murcia, La Rioja and Madrid), known as the common regime communities, only assumed responsibility for healthcare management. It was not until the period after 2007 that, through the reform of their Statutes, they were placed on an equal level with the historical autonomous communities and acquired the capacity to legislate.

Law 21/2001, of December 27, which created the new regional financing model and introduced fiscal co-responsibility, was crucial in this process. This meant the end of designated health financing. Thus, universal healthcare in Spain, instituted in 1989, is financed through taxes (that is, health financing was integrated into the general autonomous financial system without distinction from other responsibilities). In addition, the central government, through the general state budget, transfers funds to the autonomous communities, using population as a criterion for distribution, adjusted for the population over 65 years of age and insularity. Consequently, the economic resources of each autonomous community depend on the proportion of the transfers received from the central government and on the taxes assigned to the autonomous communities. Therefore, different allocated resources generate different degrees of regional financial sufficiency, which will affect particular spending policies. Indeed, the political decentralization recognized by the Spanish Constitution entails its own financial and legislative capacity, which gives rise to an inevitable regional diversity (41).

The Spanish healthcare system covers all healthcare except dental care and optical items. Medications are not free of charge, except for those dispensed during hospital admissions, or those needed for the treatment of occupational diseases. This is intended to prevent substance abuse and self-medication. However, pensioners pay a symbolic price, with a maximum of 18 euros per month. The rest of the population pays a higher or lower percentage, depending on their income level (50% for those with incomes below 22,000 euros per year, 60% for incomes between 22,000 and 100,000 euros per year, and coverage of only 18 euros for higher income levels).

In brief, Spain is considered to have one of the best, if not the best, public health systems in the world, and although it is decentralized, the autonomous communities must guarantee the universality of benefits. However, they are free to choose the method of managing the resources to achieve not only the aforementioned objective of universality but also the preservation of health and promotion of well-being, thus reducing the mortality risk (42, 43). Initially, it could be thought that decentralization favors well-being because decision-makers are closer to citizens and assumed to better understand their preferences and needs.

Summarizing, in light of the above considerations, this research has a double objective. The first is to test the research hypothesis that posits potential complex relationships between well-being and the constructs that are assumed to influence it (especially those related to public health spending), and also to estimate these direct and indirect impacts. The second is the clustering of the Spanish autonomous communities according to their indicators for well-being and the aforementioned constructs, thereby visualizing the intra-country well-being disparities due to regional public health spending policies.

The first objective is addressed by applying partial least squares structural equation modeling (PLS-SEM). This statistical method enables the simultaneous analysis of relationships between observable and latent variables and between latent variables. As such, it can deal with complex causal relationships that traditional linear regression models cannot (44). For the second objective, we used agglomerative hierarchical cluster analysis (AHCA). In addition, principal component analysis (PCA) was used to reduce the dimensionality of the problem and obtain a regional efficiency map for well-being, that is, a graphical representation of the differences between regions in terms of the two principal components that jointly capture the largest percentage of the variance in the indicators for the constructs used in this research.

The relationship between public health expenditure and well-being has not been extensively studied, despite its great importance for socio-economic agents and citizens in general, from a myriad of perspectives (health, economic, fiscal, psychological, sociological…). There is some scientific research using life expectancy as a proxy for well-being in Europe, Australia, Malaysia, sub-Saharan Africa, and Bangladesh. The results obtained are not conclusive: whereas in Eastern Europe (45), Australia (46), Malaysia (47) and sub-Saharan Africa (48) a significant relationship has been found, in Bangladesh (49) and the European Union (50) such a relationship does not exist or is marginal. The methodologies used in the above research are diverse, but traditional (data envelopment analysis, multivariate logistic regression, multivariate logistic regression, multiple linear regression with panel data and fixed effects, etc.). As far as we know, PLS-SEM has not been applied in this type of research involving complex direct, indirect, and second-order relationships between observable and latent variables in scientific research on well-being and healthcare expenditure public policies with the focus on Spain. In addition, this study is the first to explore intra-country differences in well-being arising from the different public expenditure policies implemented by the Spanish autonomous communities; this is the first time that these autonomous communities have been clustered according to the indicators for the constructs involved in the relationship between public health expenditure and well-being.

Therefore, this research contributes to filling an important gap in the literature on such a critical topic, especially in Spain, a benchmark country at the top of life expectancy rankings, which is recognized as having one of the best health public systems in the world. A secondary contribution is of a methodological nature: we successfully import PLS-SEM from other disciplines where it is widely used (marketing, tourism, hospitality management, etc.) to the vital area of healthcare.

After this introductory section, Section 2 states the hypotheses to be tested. Section 3 briefly describes the database and the statistical methodology, and Section 4 presents the results obtained. Section 5 discusses those results and concludes.

## Research hypotheses

The methodological approach used to study the direct and indirect relationships between public health spending and well-being, as well as other secondary relationships, strongly depends on both the theoretical framework underpinning the topic and on the situation and state of the art in the study area. Therefore, in this section we focus on those matters and formulate the research hypotheses accordingly.

We decided to use life expectancy at birth as a proxy variable for well-being. Fortunately, as outlined in the introductory section, Spain leads the European ranking, jointly with Switzerland, and has one of the highest life expectancy values in the world (especially for women).

Spain has an aging problem (25), which is set to worsen in the near future. According to the 2020–2070 population projections from the Spanish Statistical Office, in 2035 the population aged 65 and over will account for 26.5% of the total population (51). Obviously, population aging implies the need for more health services; in addition, when life expectancy is very high, as is the case in Spain, new health services are required in order to tackle the new (and usually chronic) pathologies typical of very elderly people (52).

Accordingly, it is no surprise that, despite the high quality public health systems the Spanish citizens enjoy, they are continuously demanding more and more, and better and better, health services. This translates into an increase in the public health spending targeted at augmenting resources, with the assumption that the more resources the higher the life expectancy. In fact, some studies claim that the 2008 crisis^1^ triggered a decline in health budgets in many European countries (53, 54) and that Spain was no exception in 2011. Compared to 2010, the budgets of the regions decreased by an average of 4.11%. In 2012, the health budget was cut by 14% (55) and 12% in 2013, albeit unevenly between regions (56). This research line claims that these reductions in public health spending may have been the cause of the poor outcome in the current health crisis caused by the SARS-CoV-2 virus (57). In contrast, there is another approach to this question that does not find a relationship between higher public health spending and better results (58–60). From this perspective, the focus should not be on the amount of expenditure but on its proper use, that is, the resources in which it is invested and the degree of use of those resources. For the researchers taking this line, health spending must be allocated to direct investment in, for example, technological medical equipment or improving facilities such as hospital beds, operating rooms, or day hospital places, among others. Note that the total budget can be used for current expenditure or investment. For nomenclature purposes, the total health budget for a given period, to be spent in that period, will be referred to as “expenditure” (or “spending”). The expenditure realized in previous years is the main driver of what we term “resources”: primary care medical staff, medical personnel in specialized care, hospital beds, operating theaters, day hospital places, technological equipment (for example, nuclear magnetic resonance or computed axial tomography equipment). Thus, current resources depend not only on current spending, but mainly on expenditure in previous periods. And, depending on its allocation, a given amount of spending may generate more or less resources for the present and the future.

The aging of the population and rising technological costs, combined with limited healthcare budgets, have put the spotlight on the sustainability of healthcare systems. Present needs must be met without threatening the possibility of meeting future needs. In this way, it will be possible to steadily reduce mortality and morbidity rates and achieve greater well-being (61). Numerous empirical studies in the literature reveal how higher healthcare spending leads to lower mortality rates. For example, local studies in India (62) or sub-Saharan Africa (63) have shown that spending reduces infant mortality. Similarly, Owusu et al., analyzing the period 2000–2015 in 177 countries, showed that expenditure led to a reduction in maternal and infant mortality (64). In this vein, a 2012–2014 study of 1,558 patients at Mount Sinai Medical Center and Icahn School of Medicine at Mount Sinai in New York City concluded that the higher the expenditure the lower the in-hospital mortality (65). Another noteworthy study, carried out by Ades et al. on 27 European Union countries, evidenced a decrease in cancer mortality related to an increase in spending (66). Therefore, we expect to find the same relationship in Spain.

Healthcare expenditure is affected by the cost of increasingly advanced technology and by the price of services, but fundamentally by the level of use of the system; for example, by the propensity to hospitalize or the induced demand (56). Therefore, strategies to reduce hospital stays, increase outpatient surgeries or even eliminate preoperative tests for those patients without a history of risk are some of the proposals made by researchers in this regard (56, 67). However, physicians should not be prevented by the system from prescribing expensive diagnostic tests or treatments, as long as they are necessary for the patient's health. In this regard, the European Federation of Internal Medicine and the American Council of Internal Medicine developed the Charter of Professionalism, which calls for medical personnel to strive for an efficient and rational use of healthcare resources, while always providing the patient with quality and safe care (68). Optimal use of resources will help to ensure a quality healthcare system (69).

On the other hand, it could be said a priori that the non-use of the health system brings health complications and can even cause death. The whole world has witnessed this phenomenon during the pandemic that started in China at the end of 2019. Health systems were severely affected: scheduled surgeries had to be canceled (70, 71); consultation with medical specialists became a challenge (72); online medical consultations rather than face-to-face were highly recommended (if not compulsory) (73); and preventive procedures for different diseases, including dangerous ones such as cancer, for example, were practically eliminated (74, 75). The latter was particularly detrimental for citizens because a delay in diagnosing diseases notably increases the risk of mortality and morbidity (75, 76). Similarly, the empirical evidence shows that neither the training of health professionals nor investment in technology are a priority for governments during periods of crisis (54, 77).

Previous studies found that countries with higher gross domestic product (GDP) had better life expectancy rates (78). That is not only due to the health system's adequate or inadequate performance but is also explained by social and economic factors such as poverty (79) or lifestyle (type of diet; sedentary lifestyle vs. physical activity; smoking, etc.) (80). In recent decades, well-being gains stem from income growth that has brought about profound transformations in living conditions (81). For this reason, we incorporate GDP as a control variable, a global indicator that reflects the added value produced by a country.

Based on the above discussion, we formulate the following research hypotheses:

Hypothesis 1 (H1): *Expenditure positively influences Resources*.
Hypothesis 2 (H2): *Expenditure negatively influences Mortality*.
Hypothesis 3 (H3): *Resources positively influence Use*.
Hypothesis 4 (H4): *Resources positively influence Well-being*.
Hypothesis 5 (H5): *Resources negatively influence Mortality*.
Hypothesis 6 (H6): *Use positively influences Well-being*.
Hypothesis 7 (H7): *Use negatively influences Mortality*.
Hypothesis 8 (H8): *Use positively influences Safety*
Hypothesis 9 (H9): *Safety negatively influences Well-being*.
Hypothesis 10 (H10): *Mortality negatively influences Well-being*
Hypothesis 11 (H11): *GDP positively influences Well-being*.
Hypothesis 12 (H12): *Safety and Mortality mediate the relationship between Use and Well-being*.
Hypothesis 13 (H13): *Mortality and Use mediate the relationship between Resources and Well-being*.
Hypothesis 14 (H14): *Resources and Use mediate the relationship between Expenditure and Mortality*.
Hypothesis 15 (H15): *Use mediates the relationship between Resources and Mortality*.

## Data and methodology

### Sample and data collection

The data used in this article, basically the key indicators of the Spanish Health System (SHS), were taken from the SHS statistical site. Obviously, this set of indicators follows the guidelines of the European Core Health Indicators (ECHI) (originally called European Community Health Indicators) developed by the European Commission to provide comparable information on European healthcare systems, and it also reflects the OECD and WHO approaches (82, 83).

In terms of spatial coverage, the statistical site of the SHS provides the annual average value of the key indicators not only at national level but also at a regional scale, that is, for the 17 autonomous communities and the two autonomous cities that make up Spain. However, these two autonomous cities have not been included in the analysis because the data on health expenditure were not available for them. In terms of temporal coverage, the period under study is 2005 to 2018, the year just before the COVID pandemic. The statistical site of the SHS provides statistical information dating back to 1990; however, it is only since 2005 that it has provided data for all the indicators we use in our PLS-SEM.

Accordingly, our database is composed of 17 (spatial dimension) x 14 (time dimension) x 26 (number of indicators, see subsection 3.2) values. That is a total of 6,188 observations. The sample size is a core factor when implementing PLS-SEM^2^ because an insufficient sample size could mean core effects or relationships existing in the population are not revealed (the well-known probability of type II error in a testing procedure with fixed significance level or probability of type I error). G^*^Power statistical software (v. 3.1.9.6) (84) can be used to determine the statistical power (or its complementary, the probability of type II error) for different model configurations and values of the linear coefficient of determination and significance level.

In our case, the number of observations per indicator (238) is large enough to consistently perform a PLS-SEM-based analysis: significance level of 0.01; mean effect of 0.15; statistical power of 0.95; minimum linear coefficient of determination (R2) in the model of 0.10. The minima suggested for the more exacting recent methods by Kock and Hadaya (85) —the inverse square root method and the gamma-exponential methods— are also exceeded when increasing the significance level to 0.05 and reducing the statistical power to 0.8.

### Variables

The variables under study are not directly observable, so they constitute what is known as constructs, composites, or latent variables. These constructs are measured (or proxied) through indicators or manifest variables used as statistical inputs to analyze their complex relationships (86). Table 1 lists and describes the six constructs (*Well-being, Mortality, Expenditure, Resources, Use* and *Safety*) and a control variable (*Economic driver*) used in this study, as well as their corresponding 26 indicators and the control variable GDP.

**Table 1:** Constructs and description of indicators.

**Construct**	**Indicator**	**Description**
Well-being	WE1	Life expectancy at birth
(mode A)	WE2	Life expectancy at age 65
Mortality	MO1	The age-adjusted death rate from cancer per 100,000 population
(mode B)	MO2	The age-adjusted death rate from cerebrovascular disease per 100,000 population
	MO3	The age-adjusted mortality rate for diabetes mellitus per 100,000 population
Expenditure	EX1	Public health expenditure managed by the autonomous communities, per inhabitant
(mode B)	EX2	Percentage of spending on specialty care services
	EX3	Percentage of public health expenditure on staff remuneration for the training of residents
	EX4	Percentage of pharmaceutical spending
Resources	RE1	Medical personnel in specialized care per 1,000 inhabitants
(mode B)	RE2	Primary care medical staff per 1,000 people assigned
	RE3	Hospital beds per 1,000 inhabitants
	RE4	Operating theaters per 100,000 inhabitants
	RE5	Day hospital places per 1,000 inhabitants
	RE6	Operating computed axial tomography (CT) equipment per 100,000 inhabitants
	RE7	Nuclear magnetic resonance (NMR) equipment per 100,000 inhabitants
Use	US1	Yearly hospital admissions per 1,000 inhabitants
(mode B)	US2	Average length of stay in hospital (in days)
	US3	Outpatient surgery percentage
	US4	Surgical intervention rate per 1,000 inhabitants/year
	US5	CT usage rate per 1,000 inhabitants/year
	US6	NMR usage rate per 1,000 inhabitants/year
Safety	SA1	Overall in-hospital mortality per 100 hospital discharges
(mode B)	SA2	In-hospital mortality post-surgery per 100 surgical discharges
	SA3	Rate of suspected adverse drug reactions
Economic driver	ED1	Gross domestic product per capita

*Well-being* was measured through life expectancy at birth (WE1) and life expectancy at age 65 (WE2). It is worth noting that Well-being is the only construct measured reflectively (mode A) because the indicators for it are competitive and represent manifestations of the construct; in other words, the causal relationship goes from the construct to the indicators and a change in the construct will immediately impact all its indicators, which translates into a strong correlation among the indicators. The other five constructs were considered as formative constructs, that is, they were measured in a formative mode (mode B), as their indicators are assumed to represent specific dimensions of the construct; accordingly, the causal relationship goes from the indicators to the construct and the indicators should not be strongly correlated (87).

Mortality rates per 100,000 population from cancer (MO1), cerebrovascular disease (MO2) and diabetes mellitus (MO3) are among the 15 leading causes of death in 2018 in Spain, accounting for 34% of the total number of deaths. Therefore, they can be considered as reliable measures of the *Mortality* construct, which is a leading referent for geographical comparisons (88).

As for the third construct, *Expenditure*, it was measured by four indicators: Public health expenditure per capita (EX1), and the percentage of spending on specialized medical care (EX2), remuneration of resident doctors in training (EX3) and medicines (EX4). It is worth noting that the Spanish Constitution of 1978 establishes that the public authorities guarantee citizens will be provided with a public Social Security System, which covers the protection of health and health services. It also makes it possible for the autonomous communities to assume responsibility in the field of health (in fact, the 17 Spanish autonomous communities have done so). In this sense, the General Health Law of 1986 establishes the political decentralization of health so that health expenditure and its distribution is different throughout the Spanish territory. This is why one of the key objectives of this study is to test for well-being disparities within the country due to the diverse public health spending policies.

The amount of current and past expenditure determines the current volume of health resources of the SHS in each geographical area, which is assumed to be an important determinant of people's well-being. *Resources* were measured with the following indicators: Number of specialty doctors (RE1), number of primary care doctors (RE2), number of hospital beds (RE3), number of operating theaters (RE4), number of day hospital places (RE5), and computer axial tomography (CT) (RE6) and nuclear magnetic resonance (NMR) (RE7) equipment.

The level of use of these resources is also considered in the literature on the topic as a driver of the population's well-being. *Use* was measured through the number of hospital admissions (US1), the average hospital stay (US2), the percentage of outpatient surgery (US3), the number of surgical interventions (US4), as well as the use of CT (US5) and NMR (US6) equipment.

The last construct considered is *Safety*, which was measured through three indicators: in-hospital mortality (SA1), mortality after surgery (SA2) and adverse medication reaction (SA3).

In addition, we include in the model a control variable, *Economic driver*, representative of the economic level of the Spanish autonomous communities, considering that a prosperous financial situation should favor well-being. It was proxied by a single indicator: GDP per capita.

### Methodology

Three different methods have been applied to test the relationship between health expenditure and population well-being.

First, the proposed model is analyzed by applying PLS-SEM (89) with the statistical software SmartPLS (v. 3.3.2.) (90). The algorithm used is the traditional PLS, i.e., composite-based (91), and path-weighting scheme (92) with a maximum number of iterations of 300 (93). PLS-SEM uses the non-parametric technique of bootstrapping with replacement to test the significance of the regression coefficients (94–96). In the first step, the measurement model, i.e., the relationships between the constructs and their indicators, is assessed. For reflective constructs, the reliability of the indicators, the reliability of the construct, and its convergent and discriminant validity are tested. For formative constructs, however, the multicollinearity between the various indicators and the significance and relevance of each indicator is analyzed. In the second step, the relationships between the constructs, known as structural modeling, are examined, which requires testing for the absence of collinearity between the latent variables as well as the magnitude, sign and significance of their connections (97).

**Figure 1:**
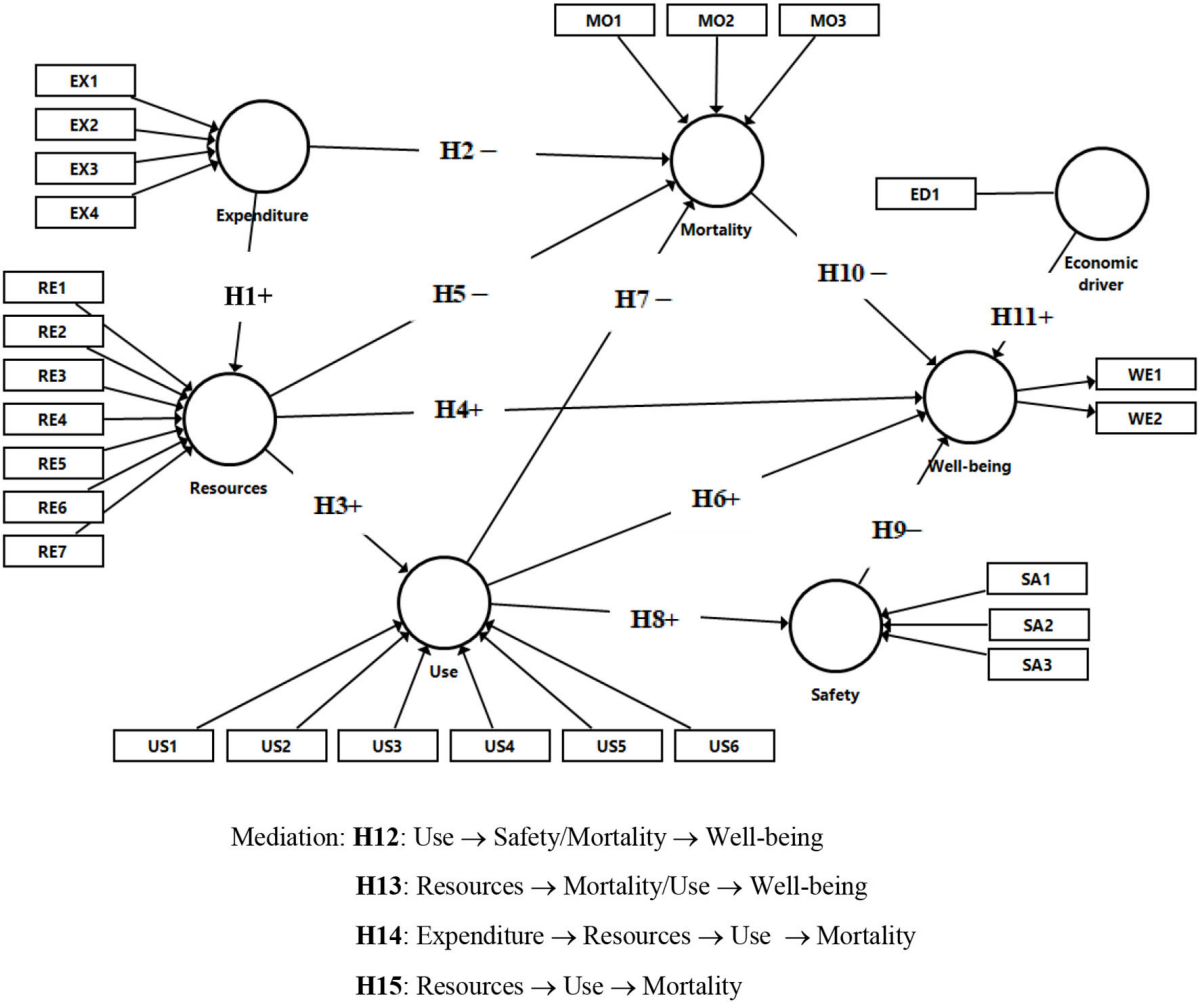
Research model.

Figure 1 shows the proposed model, which includes the constructs, indicators and research hypotheses mentioned previously.

Second, AHCA is implemented to classify the autonomous communities into groups (clusters). The territories are gradually grouped into categories that include homogeneous elements according to the variables considered and that differ from the rest of the clusters. The criterion used to establish the classification was Ward's method, which minimizes the squared Euclidean distance. In this way, the set of variables considered in the study produces a small number of regional clusters (98–100). The results of this analysis are presented, as usual, in a dendrogram.

Third, by performing a PCA, the set of variables in the study was reduced to only two dimensions, in order to provide a graphical representation that visually shows the differences between autonomous communities stemming from the inputs considered (101–103).

For those not familiar with clustering and PCA, see Bezdek (104, 105), respectively. Both statistical techniques were implemented with the software IBM SPSS Statistics 27.0 (106).

## Results

### Descriptive analysis

Table 2 reports the main descriptive statistics of the indicators or manifest variables considered in the study. In addition, Appendix 1 lists their means at a regional scale. It is worth noting the disparities between the autonomous communities regardless of the indicator. However, there is an unquestionable fact: Madrid is the autonomous community that registers the best values in all the indicators for well-being and mortality along with the lowest public health expenditure per inhabitant.

**Table 2:** Descriptive statistics.

**Construct**	**Indicator**	**Mean**	**Std**.	**Min**.	**Max**.
Well-being	WE1	82.457	1.241	78.880	85.430
	WE2	20.909	0.920	17.980	23.140
Mortality	MO1	150.192	10.834	118.330	178.560
	MO2	32.184	8.902	15.950	69.700
	MO3	11.289	5.841	2.590	42.200
Expenditure	EX1	1,412.224	164.689	1,022.620	1,876.750
	EX2	59.111	4.861	43.540	70.950
	EX3	3.284	0.922	1.260	5.870
	EX4	18.440	3.005	12.040	28.020
Resources	RE1	1.700	0.219	1.234	2.246
	RE2	0.778	0.105	0.590	1.110
	RE3	2.493	0.460	1.650	3.697
	RE4	6.465	1.015	4.300	9.037
	RE5	0.278	0.128	0.080	0.709
	RE6	1.141	0.258	0.640	1.875
	RE7	0.566	0.223	0.120	1.029
Use	US1	91.980	15.588	55.549	129.986
	US2	7.184	0.796	5.700	9.980
	US3	40.877	8.110	17.260	58.180
	US4	69.997	14.704	36.804	118.193
	US5	73.019	17.184	21.639	118.950
	US6	28.897	14.735	6.018	81.146
Safety	SA1	4.365	0.662	2.980	5.920
	SA2	1.648	0.285	0.930	2.370
	SA3	452.028	387.987	10.000	2,076.360
Economic driver	ED1	22.913	4.582	14.194	35.041

Number of observations per indicator: 238.

Going into some detail, but without intending to be exhaustive, Madrid clearly leads the way in Well-being as measured by life expectancy related indicators, WE1 and WE2; it is followed by Navarra and Castilla y León; Andalucía, Canarias and Extremadura, are the Spanish regions with the lowest life expectancy, both at birth and at 65 years of age.

Regarding Mortality indicators, again Madrid registers the best values in all of them. In MO1 it is accompanied by Castilla-La Mancha and Murcia, in MO2 by Asturias, and in MO3 by Cantabria and Galicia. País Vasco, Comunidad Valenciana and Galicia have the worst MO1 values; Andalucía, Murcia and Extremadura show the lowest values for MO2 and Canarias almost triples the national mean in MO3. As for Expenditure, interestingly, Madrid, together with Andalucía, is the Spanish region with the lowest EX1 (just over 1,200 euros per inhabitant), although there is a relevant difference between these two regions: Madrid is at the top of the Well-being ranking whereas Andalucía is at the bottom. País Vasco, Navarra, Extremadura and Asturias, in this order, are the regions with highest EX1 (more than 1,500 euro/inhabitant). It is worth noting that the public health expenditure per inhabitant managed by País Vasco is 26.3%, higher than that managed by Madrid. However, Well-being indicators in Madrid are clearly better than in País Vasco. EX2 and EX3 are led by Madrid, in the first case accompanied by Asturias and in the second by Cantabria.

On the contrary, Madrid, together with Baleares, is the region with the lowest percentage of pharmaceutical spending. Castilla-La Mancha and Castilla y León show the lowest EX2 values, País Vasco and Baleares register the lowest EX3 values, and Galicia and Comunidad Valenciana have the highest percentage of pharmacy spending. In brief, Madrid has the best Well-being results along with the lowest public health expenditure per inhabitant. Note that Madrid has the highest control over pharmaceutical spending, while showing the highest values in the country both in percentage of spending on specialty care services and on staff remuneration for residents training.

As for Resources, in general, Andalucía, Baleares and Canarias show the lowest values, whereas Aragón, Asturias, Cataluña and Extremadura are at the top of the ranking in at least two indicators. Importantly, Madrid is generally among the Spanish regions with the lowest values in Resources indicators (especially in RE2 and RE6); however, it is at the top of the ranking, together with La Rioja, in RE7 (nuclear magnetic resonance equipment per 100,000 inhabitants).

Regarding the Use of such resources, País Vasco, La Rioja and Asturias, in this order, are at the top of the ranking for US1, with Madrid at the bottom of the ranking. Canarias and Galicia are the Spanish regions with highest US2 values, whereas Cataluña and Comunidad Valenciana occupy the two last positions. La Rioja is the autonomous community with the largest outpatient surgery percentage (US3) and Navarra and Canarias the two regions with the lowest percentage. Cataluña and País Vasco have the highest values in US4, whereas Canarias exhibits the lowest value (the surgical intervention rate per 1,000 inhabitants/year is less than half that of Cataluña and País Vasco). As for usage of equipment, Galicia and Asturias lead the ranking in CT usage, while Madrid and Comunidad Valenciana are in the top two positions for NMR. At the other extreme, Baleares and Canarias, and País Vasco and Canarias, are in the last two positions of the rankings for CT and NMR, respectively

Finally, as for Safety, the ranking of SA1 is led by Baleares and Cataluña, with Galicia and Asturias in the last two positions. Navarra has the best (lowest) SA2 ratio, and Galicia and Cantabria the worst ones. Navarra, Asturias and Aragon, in this order, have the highest rate of suspected adverse medication reactions (SA3), almost five times that of Castilla-La Mancha, which has the lowest rate.

Regarding the control variable GDP per capita, the ranking is led by Madrid, followed by País Vasco and Navarra. The bottom three positions are occupied, in this order, by Castilla-La Mancha, Andalucía, and last of all, Extremadura.

In brief, as mentioned previously, there are important disparities among the Spanish autonomous communities regardless of the indicator. Nevertheless, there seem to be important political (the sign of the government, when this sign has been the same for a long time; this is the case of Madrid, Andalucía and Extremadura), geographical (in the case of Canarias) and historical-political (in the case of País Vasco and Navarra) latent reasons behind such disparities.

### Partial least squares structural equation modeling

**Figure 2:**
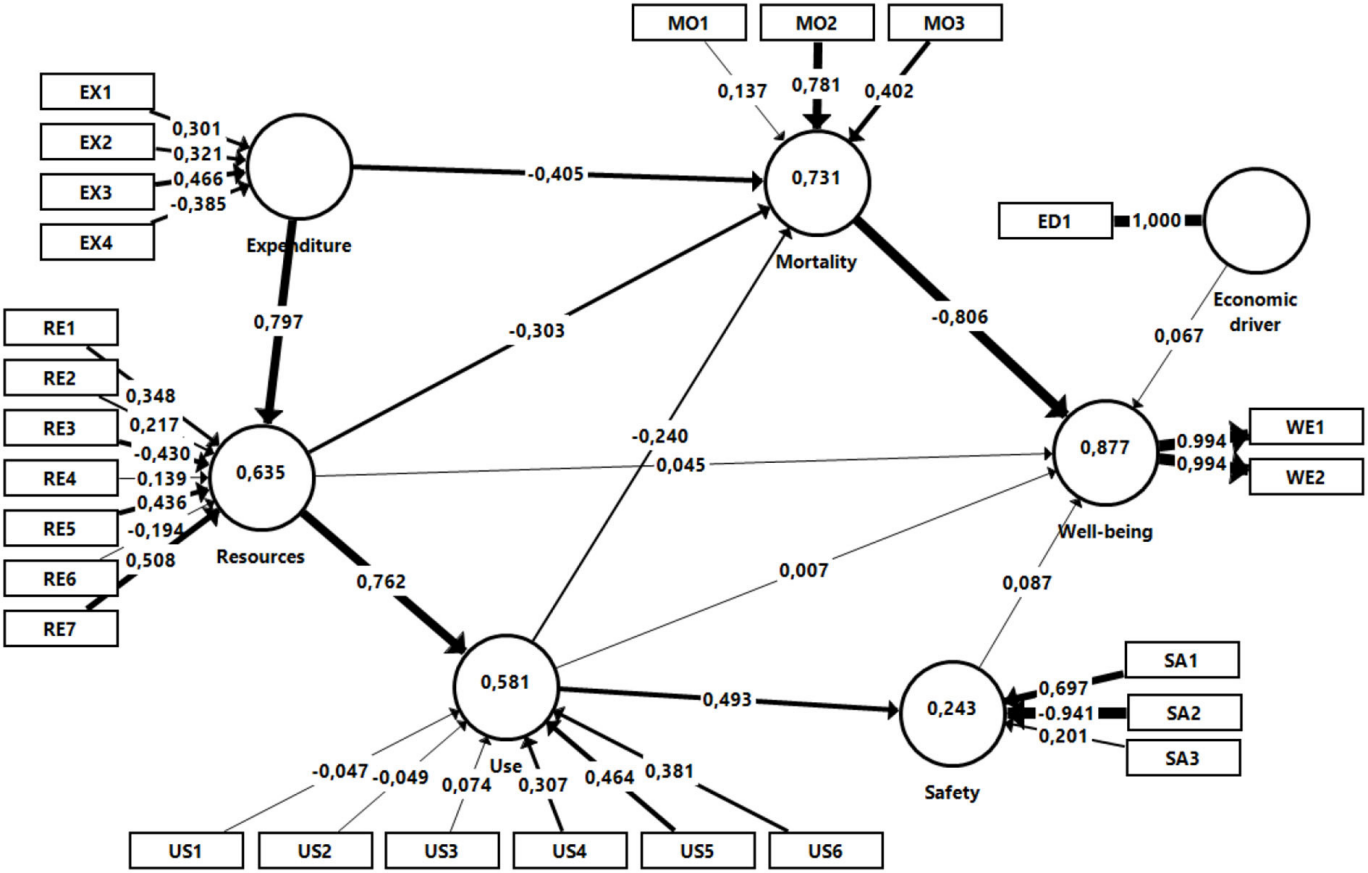
Model results.

Figure 2 shows both the inner and outer estimation results for the PLS-SEM specification we propose to test the research hypotheses listed in Section 2.

#### Measurement model

Table 3 reports the results on the validity of Well-being, the only latent variable considered in reflective mode (mode A). The construct is acceptable since all indicator loads are >0.707 (Panel A) (107). Moreover, the construct reliability is verified, given that Cronbach's Alpha, Dijkstra-Henseler's Rho and Composite Reliability are >0.7 (91, 108). The average variance extracted (AVE) exceeds 0.5, supporting the convergent validity (109). Specifically, Well-being explains, on average, 98.8% of the variance in its two indicators. Finally, the Fornell-Larcker criterion supports the discriminant validity (110) since 0.994 (marked in bold on the diagonal), which is the square root of the AVE, is greater than the correlation of Well-being with the rest of the latent variables (values located on the same horizontal line). Likewise, the heterotrait-monotrait ratio (HTMT) (111), 0.526, indicates that Well-being is a discriminant construct since this value is < 0.85.

**Table 3:** Outer model evaluation. Reflective construct-mode A (well-being).

**Indicator**	**Load (λ)**	***p*-value**	**CI 2.5%**	**CI 97.5%**			
**Panel A. Outer loads**							
WE1	0.994^**^	0.000	0.992	0.995			
WE2	0.994^**^	0.000	0.992	0.995			
	**Value**	***p*** **-value**	**CI 2.5%**	**CI 97.5%**			
**Panel B. Construct reliability and average variance extracted**							
Cronbach's Alpha	0.988^**^	0.000	0.984	0.991			
Dijkstra–Henseler's Rho	0.988^**^	0.000	0.985	0.991			
Composite reliability	0.994^**^	0.000	0.992	0.995			
AVE	0.988^**^	0.000	0.985	0.991			
	**ED**	**EX**	**MO**	**RE**	**SA**	**US**	**WE**
**Panel C. Discriminant validity (Fornell-Larcker criterion)**							
Expenditure (EX)	n.a.						
Mortality (MO)	−0.511	n.a.					
Resources (RE)	0.343	0.797	n.a.				
Safety (SA)	0.292	0.495	−0.558	n.a.			
Use (US)	0.295	0.570	−0.702	0.762	n.a.		
Well-being (WE)	0.523	0.740	−0.931	0.774	0.585	0.671	**0.994**
	**Value**	**Mean**	**CI 2.5%**	**CI 97.5%**			
**Panel D. discriminant validity (HTMT criterion)**							
WE → ED	0.526	0.525	0.433	0.607			

Two-tailed test.

* Significant at 5% significance level;

** Significant at 1% significance level. The significance of the loads and their 95% confidence interval were calculated by a bootstrapping procedure with 10,000 replications.

Table 4 includes the data corresponding to the assessment of the structural measurement model of the constructs estimated in formative mode (mode B). As can be seen, there are no multicollinearity problems (VIF < 5), and all indicators were kept in the model since those whose weights were not significant did exhibit significant loads (97, 112).

**Table 4:** Assessment of the measurement model. Formative constructs-mode B.

	**VIF**	**Weight**	***p*-value**	**CI 2.5%**	**CI 97.5%**	**Load**
**Mortality**						
MO1	1.341	0.137^**^	0.000	0.078	0.196	0.598^**^
MO2	1.303	0.781^**^	0.000	0.719	0.837	0.900^**^
MO3	1.049	0.402^**^	0.000	0.318	0.486	0.535^**^
**Expenditure**						
EX1	1.263	0.301^**^	0.000	0.178	0.419	0.445^**^
EX2	2.199	0.321^**^	0.000	0.178	0.445	0.807^**^
EX3	1.302	0.466^**^	0.000	0.358	0.561	0.649^**^
EX4	2.145	−0.385^**^	0.000	−0.503	−0.276	−0.791^**^
**Resources**						
RE1	2.045	0.348^**^	0.000	0.242	0.453	0.708^**^
RE2	1.367	0.217^**^	0.000	0.130	0.300	0.204^**^
RE3	1.541	−0.430^**^	0.000	−0.532	−0.322	0.113*^*ns*^*
RE4	3.317	0.139*^*ns*^*	0.062	−0.004	0.287	0.644^**^
RE5	1.737	0.436^**^	0.000	0.337	0.537	0.768^**^
RE6	2.413	−0.194^**^	0.001	−0.303	−0.083	0.468^**^
RE7	2.113	0.508^**^	0.000	0.372	0.636	0.835^**^
**Use**						
US1	2.237	−0.047*^*ns*^*	0.645	−0.244	0.157	0.470^**^
US2	1.944	−0.049*^*ns*^*	0.604	−0.239	0.135	−0.398^**^
US3	1.694	0.074*^*ns*^*	0.350	−0.084	0.223	0.475^**^
US4	2.984	0.307^**^	0.004	0.088	0.508	0.691^**^
US5	3.026	0.464^**^	0.000	0.270	0.672	0.899^**^
US6	2.946	0.381^**^	0.000	0.167	0.579	0.887^**^
**Safety**						
SA1	1.564	0.697^**^	0.000	0.465	0.874	0.333^**^
SA2	1.542	−0.941^**^	0.000	−1.065	−0.755	−0.681^**^
SA3	1.339	0.201*	0.037	0.017	0.377	0.632^**^

Two-tailed test.

* Significant at 5% significance level;

** Significant at 1% significance level. The significance of the weights and their 95% confidence intervals, as well as the significance of the loads, were calculated by a bootstrapping procedure with 10,000 replications. ns, not significant.

#### Structural model

Table 5 shows the main results of the structural model estimation, with the signs of the coefficients assigned according to the relationships posited in the research hypotheses formulated in Section 2. The assessment was performed through one-tailed bootstrapping with 10,000 replications (113, 114). No collinearity problems were found.

**Table 5:** Assessment of the structural model. Direct and Total effects.

		**Path**	***p*-value**	**CI 5%**	**CI 95%**
**Panel A. Direct effects**						
ED → WE		0.067^**^	0.005	0.024	0.109
EX → MO		−0.405^**^	0.000	−0.501	−0.302
EX → RE		0.797^**^	0.000	0.762	0.835
MO → WE		−0.806^**^	0.000	−0.879	−0.733
RE → MO		−0.303^**^	0.000	−0.428	−0.190
RE → US		0.762^**^	0.000	0.729	0.806
RE → WE		0.045*^*ns*^*	0.143	−0.022	0.118
SA → WE		0.087*	0.013	0.029	0.141
US → MO		−0.240^**^	0.000	−0.308	−0.167
US → SA		0.493^**^	0.000	0.414	0.582
US → WE		0.007*^*ns*^*	0.419	−0.052	0.065
		**Effect**	***t***	**CI 5%**	**CI 95%**
**Panel B. Total effects**						
ED → WE		0.067^**^	0.005	0.024	0.109
EX → MO		−0.793^**^	0.000	−0.831	−0.758
EX → RE		0.797^**^	0.000	0.762	0.835
EX → SA		0.299^**^	0.000	0.249	0.367
EX → US		0.607^**^	0.000	0.568	0.661
EX → WE		0.706^**^	0.000	0.668	0.748
MO → WE		−0.807^**^	0.000	−0.879	−0.733
RE → MO		−0.486^**^	0.000	−0.588	−0.393
RE → SA		0.376^**^	0.000	0.312	0.456
RE → US		0.762^**^	0.000	0.729	0.806
RE → WE		0.476^**^	0.000	0.389	0.572
SA → WE		0.087*	0.013	0.288	0.142
US → MO		−0.240^**^	0.000	−0.308	−0.167
US → SA		0.493^**^	0.000	0.414	0.582
US → WE		0.244^**^	0.000	0.162	0.320
**Dependent variable**	*R* ^2^	**Antecedent** **variables**	**Path coefficients**	**Correlations**	**Explained variance**
**Panel C. Decomposition of the explained variance**					
Well-being	0.877	Economic driver	0.067	0.523	0.035
		Mortality	−0.806	−0.931	0.751
		Resources	0.046	0.774	0.035
		Safety	0.0878	0.585	0.051
		Use	0.007	0.671	0.005
Mortality	0.731	Expenditure	−0.405	−0.784	0.318
		Resources	−0.303	−0.809	0.245
		Use	−0.240	−0.702	0.168
Resources	0.635	Expenditure	0.797	0.797	0.635
Safety	0.243	Use	0.493	0.493	0.243
Use	0.581	Resources	0.762	0.762	0.581

One–tailed test.

* Significant at 5% significance level;

** Significant at 1% significance level. Both the significance of the path and effect coefficients, as well as their 95% confidence intervals were calculated by a bootstrapping procedure with 10,000 replications.

Panel A reveals that Expenditure significantly influences both Resources and Mortality, the former positively and the latter negatively (*p* = 0.000 in both), which supports^3^ hypotheses H1 and H2.

Likewise, Resources positively and significantly influences Use, and negatively and significantly influences Mortality (*p* = 0.000 in both cases); however, its relationship with Well-being is not significant (*p* = 0.143). Consequently, the empirical evidence from the PLS-SEM model estimated supports hypotheses H3 and H5, but not H4.

Use proved to have significant relationships with Mortality and Safety (*p* = 0.000 in both cases), but not with Well-being. The relationship with Mortality is negative, whereas with Safety it is positive. Therefore, H7 and H8 were validated, but not H6. Furthermore, Safety exerts a small (path coefficient of 0.087) although significant (*p* = 0.013) influence on Well-being, but with a different sign than expected, which leads us to reject H9.

Finally, Mortality negatively, and strongly, influences Well-being (the path coefficient is −0.806, with an associated *p*-value of 0.000), which validates H10. With respect to the control variable, Economic driver, a positive weak but significant relationship with Well-being is found, thus verifying H11.

Panel B shows that the total effects of some constructs on others are all significant. Therefore, considering that the direct effect of Use on Well-being is not significant, but the total effect is, it can be concluded that the mediation by Safety and Mortality of the relationship between Use and Well-being is a *full mediation*, verifying H12. Similarly, given that the direct effect of Resources on Well-being is not significant, but the total effect is, we can conclude that the mediation by Mortality and Use of the relationship between Resources and Well-being is again a *full mediation*, thus validating H13.

Finally, since the direct and total impacts of Expenditure on Mortality turned out to be significant, there is a *partial mediation* by the variables Resources and Use of the relationship between Expenditure and Mortality, supporting H14. Similarly, the variable Use *partially mediates* the relationship between Resources and Mortality, supporting H15.

Panel C shows the model's explanatory power through the *R*^2^ coefficient and reports the decomposition of the variance explained by the preceding constructs. The antecedent variables for Well-being explain 87.7% of the variance in Well-being under a linear relationship, with Mortality being the most influential construct. Considering that more than 70% of the variance in Mortality is explained by Expenditure and Resources and, to a lesser extent, by Use, it can be concluded that Well-being is strongly determined by these variables. Indeed, this statement can be corroborated in light of the total effects of each construct on Well-being: −0.807 for Mortality, 0.706 for Expenditure, 0.476 Resources and 0.244 Use. However, as outlined previously, Safety and Economic driver show small total effects on Well-being (0.087 and 0.067, respectively). Given the *R*^2^ values, it can be concluded that the model demonstrates a high explanatory power (112, 115). Finally, the Stone-Geisser test-statistic, with a value of 0.8594, indicates a high in-sample predictive power of the final reflective dependent construct (116).

### Agglomerative hierarchical cluster analysis

**Figure 3:**
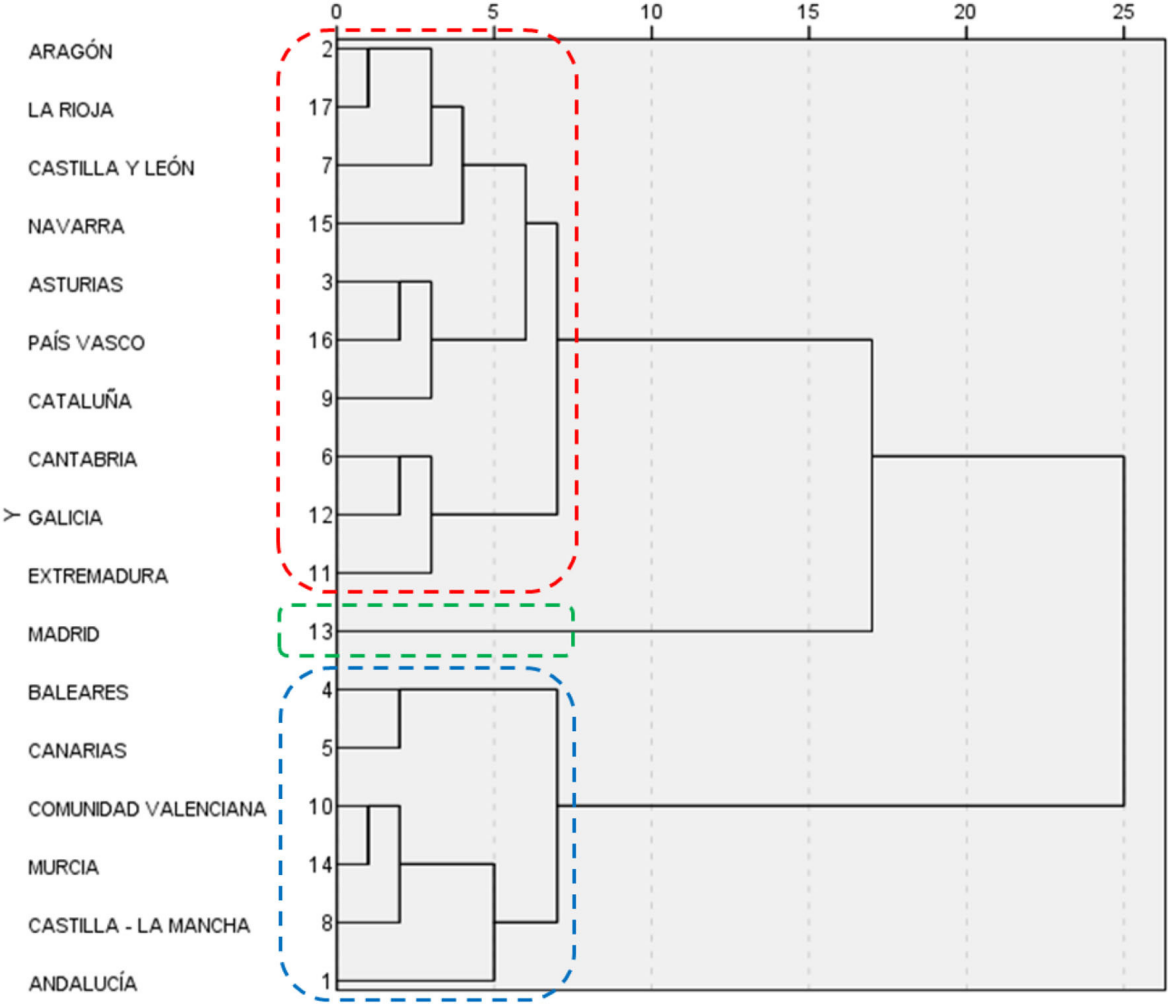
Dendrogram.

**Figure 4:**
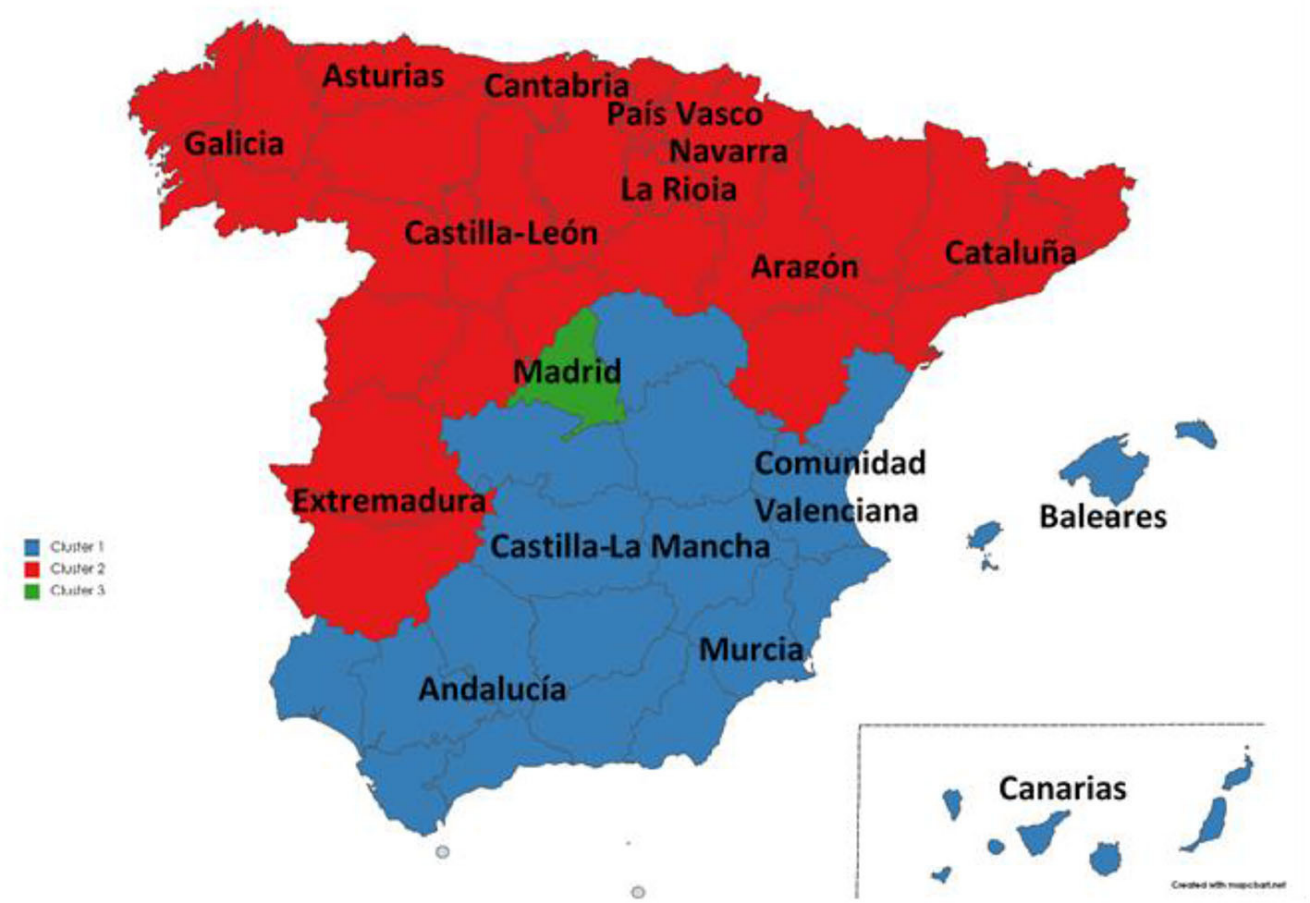
Spanish map of clusters.

Having estimated the total effects of the formative constructs and the control variable on the reflective composite, we address the second objective of this research: implementing an AHCA (Ward method) to classify the Spanish autonomous communities into groups according to the indicators of the constructs considered in this research. More specifically, in light of the results of the estimation of our PLS-SEM specification (see Figure 2), which indicates that Mortality, Expenditure and Resources are the factors that have the greatest impact on Well-being, the cluster analysis has been performed using as inputs only the indicators for the abovementioned constructs. Figure 3 depicts the resulting dendrogram. Figure 4 depicts the regional clusters on the map of Spain. Finally, the mean values of the abovementioned indicators for each cluster are shown in Appendix 2.

As can be seen in Figures 3, 4, three clusters can be clearly distinguished. Cluster 1 (in blue) is composed of Baleares, Canarias, Comunidad Valenciana, Murcia, Castilla-La Mancha and Andalucía. Shown in red is cluster 2, which comprises Aragón, La Rioja, Castilla y León, Navarra, Asturias, País Vasco, Cataluña, Cantabria, Galicia, and Extremadura. Finally, in green is cluster 3, consisting only of Madrid, which displays atypical behavior that does not fit with the rest of the country's regions.

To check that the clusters were indeed well-constructed, an ANOVA was performed in which the means of each group were used together with that of Economic driver. The goodness of fit for this ANOVA model (*F* = 5.237 and *p*-value = 0.020) supports the results obtained from the dendrogram shown in Figure 3.

As shown in Appendix 2, the first cluster (Baleares, Canarias, Comunidad Valenciana, Murcia, Castilla-La Mancha and Andalucía) is characterized by the lowest Well-being and the highest Mortality rates. The indicators for Expenditure in this group generally lie between those of groups 2 and 3.

Group 2 has similar Well-being values to those of group 1, although the Mortality rates are the highest of the country. The Expenditure indicators show values between those of groups 1 and 3, except for E3, whose mean is close to that of group 1.

Madrid, the only member of group 3, shows the best results in Well-being and Mortality along with the lowest public health expenditure per inhabitant and percentage of pharmaceutical spending, and the highest percentage in specialty care services and medical staff spending.

There are no notable differences between the three groups in the indicators for Resources.

### Principal component analysis

Finally, a PCA was applied to the variables used in the previous stage to reduce the indicators to only two independent principal components and subsequently produce a graphic representation of the autonomous communities according to these two factors. After performing the Bartlett test of sphericity, yielding a *p*-value of 0.000, the principal components were computed, so that the first two account for 57.65% of the variance in the 16 indicators for Well-being, Mortality, Expenditure and Resources. This percentage can be considered high enough to proxy the Well-being/Healthcare reality of the Spanish autonomous communities through a two-factor map.

**Figure 5:**
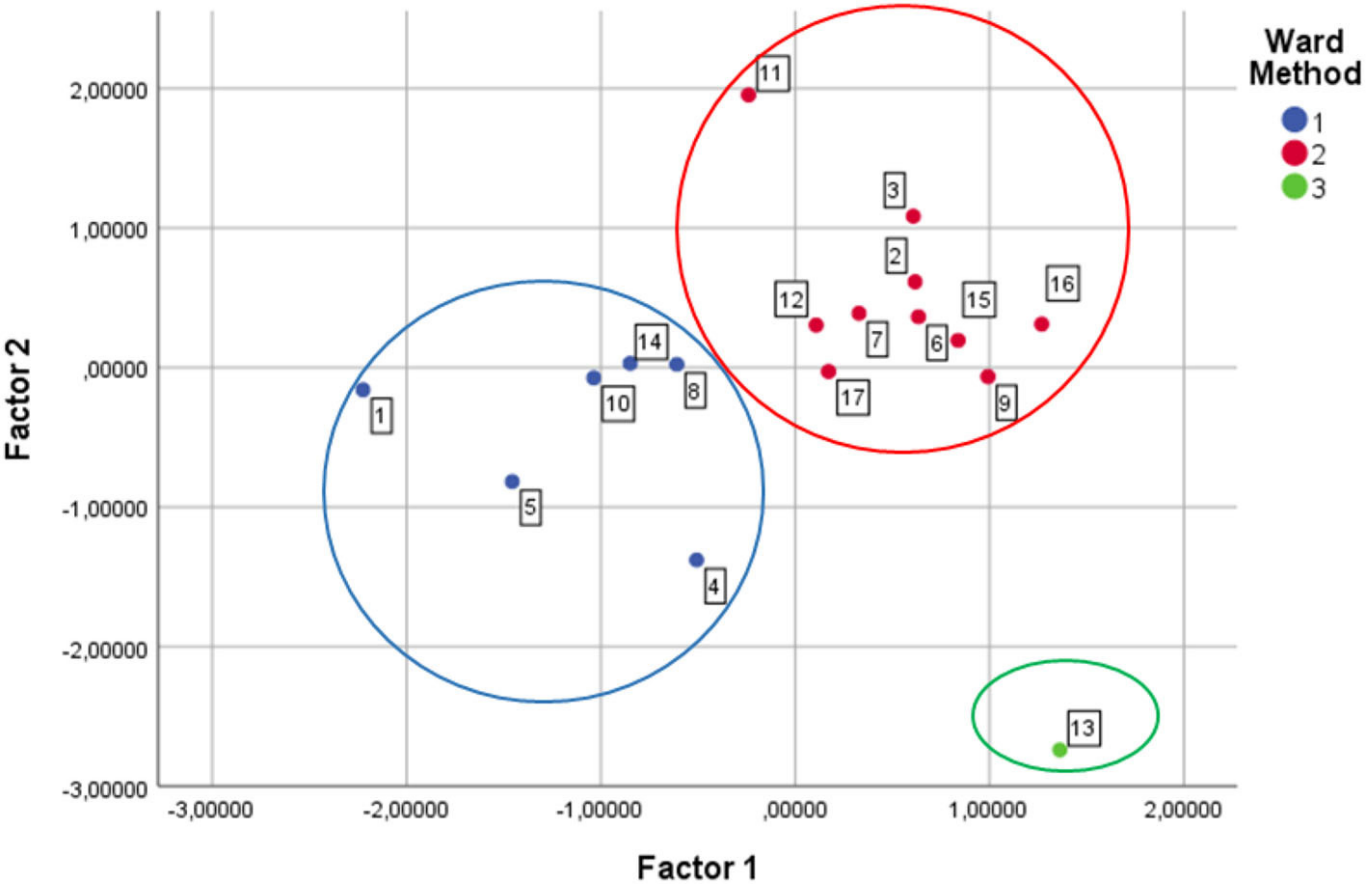
Factors chart.

Figure 5 depicts a graphical representation of the three previous clusters as a function of the first two principal components. Table 6 shows the factor loadings matrix.

**Table 6:** Factor loadings matrix.

**Variable**	**Factor 1**	**Factor 2**
RE1	0.856	0.222
RE2	0.159	0.597
RE3	0.527	0.594
RE4	0.616	0.599
RE5	0.564	0.158
RE6	0.232	0.757
RE7	0.848	0.067
EX1	0.386	0.676
EX2	0.489	−0.596
EX3	0.290	−0.403
EX4	−0.555	0.350
WE1	0.791	−0.447
WE2	0.802	−0.461
MO1	−0.012	0.744
MO2	−0.619	0.426
MO3	−0.628	0.089

Extraction method: principal component analysis.

According to the entries of the factor loadings matrix, the first principal component (factor 1) is associated with lower mortality, higher life expectancy, higher percentage of spending on specialist physicians and, consequently, more specialist physicians, more places in day hospitals and more NMR equipment. By contrast, factor 2 is associated with more healthcare spending, a higher percentage of spending on pharmacy, more primary care physicians and more CT equipment. However, the loadings of the number of hospital beds and operating theaters are similar for both factors. Considering these results, factor 1 can be identified with “healthiness” and factor 2 can be interpreted as “basic spending”.

It can be observed that the results obtained confirm those of the cluster analysis: the autonomous communities of the blue cluster register less basic spending and are less healthy. Conversely, the communities included in the red cluster register more basic spending and, in return, they are healthier. An interesting exception is Madrid, which has the lowest basic spending, but at the same time is the healthiest region in the country.

## Discussion and conclusions

The topic of well-being inequalities arising from different healthcare expenditure public policies is a very interesting topic at a national scale, but is especially worthy of study at a sub-national level. Accordingly, we focused our research on Spain, the reasons being that (i) it is among the top countries in life expectancy rankings; (ii) it is considered to have one of the best public health systems in the world; and (iii) it has a decentralized public health system at the scale of the autonomous community. There is an additional reason: according to a celebrated study by Foreman et al. (117) based on 79 drivers for health as well as on trends in health, in Spain life expectancy at birth will reach 85.8 years in 2040, one of the highest in the world.

What is behind such a high life expectancy? The commonly proposed reasons that can be found in the literature on the topic are the absence of armed and social conflicts, the sharp decline in deaths from traffic accidents, the diet, the climatological and environmental conditions, the policies that have made it possible to improve the lifestyle of the population, the significant reduction in the infant mortality rate and, especially, the notable improvements in the medical treatments and the quality of care in the universal public health system. Therefore, in one way or another, the different aspects of healthcare expenditure public policies are behind life expectancy and, as explained below, behind well-being^4^.

As outlined in the introductory section, when approaching research on health spending, a key question is what modern societies are primarily seeking to achieve with such spending. There is a myriad of possible answers as to this overall objective; selecting one is an intellectual challenge and remains a pending task. In this research, we initially considered wellness and well-being, before eventually selecting well-being because (i) it could be said that wellness is an important element of overall (multidimensional) well-being, and that well-being is a more inclusive concept than wellness; (ii) wellness is often identified with individuals and well-being with groups of individuals; (iii) governments are currently implementing programs focused on the well-being of citizens.

Given the non-observability of the variables involved in the complex relationship between well-being and health spending (there are more hypotheses than certainties regarding this relationship), we have addressed the impacts of health spending-related constructs on well-being in the framework of a PLS-SEM specification. To proxy the abovementioned constructs, we used a set of 26 indicators. In addition, regional GDP per capita was used as a control variable.

The estimation of the PLS-SEM specification we propose yields some interesting results. From the estimates corresponding to the inner part of the model, it can be concluded that Mortality is the construct that most influences, negatively, Well-being (the path coefficient is −0.806), which validates H10. Safety also exerts a small though significant influence on Well-being, but with a different sign than expected. The control variable, Economic driver, has a weak positive relationship with Well-being. The other formative constructs have no significant influence on Well-being. Other interesting results obtained from the estimation of the model are the influence of Expenditure on both Resources and Mortality, the positive impact of Resources on Use and its negative effect on Mortality, and the significant relationship of Use with Mortality and Safety. As for second-order relationships, the analyses of direct and total effects lead us to conclude that Safety and Mortality *fully mediate* the relationship between Use and Well-being, that Mortality and Use *fully mediate* the relationship between Resources and Well-being, while the variables Resources and Use *partially mediate* the relationship between Expenditure and Mortality; similarly, Use *partially mediates* the relationship between Resources and Mortality.

Summarizing, in light of the results from the estimation of the proposed PLS-SEM specification, only the fourth, sixth and ninth research hypothesis were rejected, whereas the other twelve were supported. It is important to note that the link between quality and quantity of health funding is far from being a direct relationship, since the way in which the budget is managed plays a crucial role. Spending more does not mean spending better. Therefore, a particular allocation of resources may not be the best way to achieve better well-being, which could explain the rejection of H4, despite the fact that the total effects indicate that Resources have a positive and significant influence on Well-being. In addition, preventive medicine and social awareness policies, such as flu vaccine drives or accident-prevention campaigns, should be considered. Accordingly, there might be a link between well-being and corporate governance that would explain the failure to support H6, whereas Use directly and positively influences well-being. Furthermore, considering that Safety indicators would increase with Use, and that Use has a positive overall effect on Well-being, the positive sign of the relationship between Safety and Well-being would make sense, explaining the rejection of H9.

As for the estimates relating indicators with formative constructs in the outer part of our PLS-SEM specification, it is worth highlighting that all of them have a similarly moderate impact on Expenditure (the percentage of pharmaceutical spending in a negative sense). There is a logical explanation for the positive and significantly influence of spending on medical resident training. It is understood that having more trained professionals will enable more efficient patient care. As expected, investments in human capital have effects on social well-being (81). As for the negative sign for pharmaceutical spending, some members of the population are not willing to consume pharmaceuticals, preferring to opt for alternative medicine or treatments when possible. The propensity to take medication is significantly lower in those under 65 years of age than in older people (118). People feel uncomfortable taking medication because it interrupts their regular routine and behavior. For example, a previous study revealed that non-adherence to the consumption of medicines was explained by factors such as warnings against drinking alcohol with medication, people's inability to remember the exact time of intake or its impact on their sex life (119).

Regarding the construct Resources, the indicators that had a positive impact on it were, in this order, NMR equipment per 100,000 inhabitants, day hospital places per 1,000 inhabitants and medical personnel in specialized care per 1,000 inhabitants; and to a lesser extent, primary care medical staff per 1,000 people assigned. On the contrary, the number of hospital beds per 1,000 inhabitants, and to a lesser extent, CT equipment per 100,000 inhabitants, show a negative effect on the construct. There are a number of possible explanations for the sign of the aforementioned impacts: it is worth noting that, in Spain, NMR has a better reputation than CT because it does not produce radiation; in addition, it is more user friendly for patients. When circumstances allow it, patients prefer to continue their recovery process at home, which explains the negative impact of the number of hospital beds and the positive effect of day hospital places. These relationships are consistent with previous research (120).

In the case of the degree of Use, only three of the six indicators were significant: first of all, the CT usage rate per 1,000 inhabitants/year; in second place, the NMR usage rate per 1,000 inhabitants/year; and finally, the surgical intervention rate per 1,000 inhabitants/year, all of which exert a positive influence.

As for Mortality, it is explained by three indicators, one of which is the most influential of the entire model in a positive sense. We are referring to the age-adjusted death rate from cerebrovascular disease per 100,000 population. The strong positive relationship of this indicator with the construct is not trivial, given that, according to the 2014 WHO Report (121), cardiovascular diseases are responsible for 37% of deaths of people under 70 years of age. The age-adjusted mortality rate for diabetes mellitus per 100,000 population has a moderate positive influence on Mortality, whereas the positive effect of the age-adjusted death rate from cancer per 100,000 inhabitants is very low. The importance of early prevention in these diseases is crucial to limiting their mortality rate. In addition, diabetes brings with it other diseases; consequently, comprehensive care of patients is essential to maintain their well-being and quality of life (122).

Finally, Safety is explained by three indicators. In-hospital mortality after surgery per 100 surgical discharges is the indicator that has the greatest (negative) impact on Safety; in fact, its coefficient is close to −1. The overall in-hospital mortality per 100 hospital discharges has a strong positive effect on the construct, whereas the impact of the rate of suspected adverse medication reactions is both positive and moderate.

As for the results of the regional clustering analysis, which obviously correspond to those from the descriptive analysis (Table 2), three clusters can be clearly distinguished. Cluster 1 is composed of Baleares, Canarias, Comunidad Valenciana, Murcia, Castilla-La Mancha and Andalucía. It is characterized by the lowest Well-being and the highest Mortality rates. The indicators for Expenditure in this group generally lie between those of groups 2 and 3. Cluster 2 includes Aragón, La Rioja, Castilla y León, Navarra, Asturias, País Vasco, Cataluña, Cantabria, Galicia, and Extremadura, and it has similar Well-being values to those of cluster 1; however, it also exhibits the highest Mortality rates of the country. The spending indicators show values between those of clusters 1 and 3, except for the percentage of public health expenditure on staff remuneration for training residents, whose mean is close to that of cluster 1. Cluster 3 is a single-region cluster including only Madrid, which displays atypical behavior that does not fit in with the rest of the country's regions. Madrid shows clearly superior results in Well-being and Mortality, with the lowest public health expenditure per inhabitant and percentage of pharmaceutical spending, and the highest percentage in specialty care services and medical staff spending. Interestingly, there are no noticeable differences between the three groups in the indicators for Resources.

The Community of Madrid is the region that spends the least and ranks at the top for Well-being. This performance could be attributed to Madrid having a younger population than the rest of the regions, which would imply that they are less dependent on the health system. However, this is not the case: Madrid is in fourth position for average age by autonomous community ordered from lowest to highest, with the communities with the youngest population being the Region of Murcia, Baleares and Andalucía (123). There are a number of possible reasons for this successful result: First, that the right party, which can be said from an economic perspective to be a liberal party, has governed the Community for almost three decades. Second, that the Community of Madrid exhibits the highest population density of the country, approximately 800 inhabitants per km^2^ (124). In other areas of the country the population is more dispersed than in Madrid, and it is necessary to invest in hospitals distributed among many low-density population centers; this is the case with Castilla-La Mancha, Castilla y León, and Extremadura, where the population density is 25 inhabitants per km^2^ (124). That said, it should be noted that although in recent decades the regional governments of Madrid have made a firm commitment to a large hospital network, Madrid is not the Spanish region with the largest number of hospitals. Cataluña with 69, Andalucía with 50, and Comunidad Valenciana with 39 all have more public hospitals than Madrid (with 35, some of which enjoy global recognition for their quality). Cataluña and Andalucía also have more private hospitals than Madrid (150 and 62, respectively, whereas Madrid has 48). Similar comments can be made about the number of hospital beds. Third, Madrid, Andalucía, Cataluña and Comunidad Valenciana, in this order, are the regions with the largest endowment of high-tech equipment, although in the case of Cataluña 61% of this equipment is in private centers. The other two Spanish regions following Cataluña in the percentage of high-tech equipment located in private hospitals are Baleares (54%) and Canarias (46%). Fourth, Madrid is, after two of the smallest regions of Spain, Asturias and La Rioja, the region with the shortest waiting time for patients to be attended in consultations, and the region with the lowest rate of patients waiting per surgery per 1,000 inhabitants. Fifth, Madrid (36.6%), together with Cataluña (31.8%) and Baleares (29.5%), is one of the Spanish regions with the highest health insurance capillarity, which might allow for lower public spending while at the same time maintaining the quality of the health system (125). Data for reasons two and three were taken from Acta Sanitaria (126). UNESPA (127) is the source for data cited in reasons four and five.

However, despite the statistical information provided above, Well-being in Cataluña, and especially in Andalucía, Comunidad Valenciana and Baleares, is noticeably lower than in Madrid. These results suggest that the “Madrid model” should be explored by the rest of the Spanish autonomous communities, especially those that have a very large population and/or are densely populated.

Finally, coming back to the non-clustering-related results, it can be also concluded that the higher the per capita health expenditure, the higher the percentage of spending allocated to specialized medical personnel and the training of doctors, and the lower the pharmaceutical spending. Given the significant positive influence of Expenditure on Well-being, this translates into an increase in the Well-being of the population. Likewise, when the number of physicians, both in primary and specialized care, the number of places in day hospitals and NMR equipment increases, the life expectancy of the population also rises. Additionally, the number of surgical interventions and the use of CT and NMR equipment contribute to an increase in Well-being. Therefore, according to this research, medical personnel and advanced diagnostic equipment, as well as the freedom for doctors to resort to surgical interventions without budgetary restrictions, are core resources of the healthcare system.

The results revealed in this study are of great importance for public policymakers. As is well-known, there is a current of opinion in Spain, independent of people's political alignment, which calls for the centralization of health services. The main reason giving rise to this current of feeling is that many citizens have to travel dozens of kilometers to go to hospital when in fact their nearest hospital is in the neighboring autonomous community. In addition, more and more citizens are aware of the differences in the costs and services of the different regional health systems. Some autonomous communities are recognized as having some of the best hospitals in Europe (and in some cases the world, as in Madrid) and, unfortunately, others are known for their long waiting lists. Political battles over the size and way of managing the health budget must be based on scientific research rather than on slogans or simple opinions, both regionally and nationally. More taxes, supposedly allocated to the health system, does not necessarily imply greater Well-being. It depends on how the resources are used and where the funds are spent. On many occasions, the inappropriate management of health spending means extra expenditure does not result in a proportional increase in citizens' Well-being and life satisfaction. This is the lesson we have learned from this research, especially from the Community of Madrid. This is an important lesson for Spain's left-wing and extreme left political parties; a lesson that should encourage them to abandon the mantra that more spending necessarily implies greater Well-being. The amount of spending is a necessary but not sufficient condition for Well-being. The Spanish autonomous communities must study the modus operandi of the Madrid health system, because the Community of Madrid is, by far, the leader of the Well-being ranking while spending relatively little compared to the rest of the regions.

As for the limitations of this research, they include two that are worth mentioning. The first one is the assumption that patients in each autonomous community are treated by the health services of the community in which they reside. However, given the inevitable mobility of the population throughout the country, there are many interactions between regional health systems. For example, if a citizen from any region of the country has a problem that requires very specialized care, he or she is transferred to the big cities, especially Madrid. In this way, the largest cities provide healthcare not only to their own population, but also to patients from other regions. The same applies in tourist areas, where non-residents are treated. It was not possible to account for this fact in this study, which is a limitation of our research that could give rise to a future research line.

The second limitation is framed within the relationship between life expectancy and Well-being. A long life expectancy means living many years, which is not the same as living many good quality years. This is an important aspect to consider, especially in the case of women. As is well-known, there are physiological aspects underlying the difference in longevity between men and women. Female hormones protect against mortality after the end of reproductive age but are also a factor in the development of problems in the bones, muscles, and joints. Therefore, deflating female life expectancy using some quality-of-life index related to the aforementioned aspects can yield a more realistic relationship between life expectancy and Well-being. Finding a way to overcome this limitation represents another future challenge for researchers on this topic.

We would like to emphasize that the data used in this research correspond to the usual health situation of the country, as they do not include the years of the pandemic. They therefore do not reflect expenditure patterns or ways of dealing with times of crisis in each region. However, an interesting line of future research would be to compare the pre- and post- pandemic health situation in terms of (i) testing the research hypotheses (which posit the potential complex relationships between Well-being and the constructs that are assumed to influence it, especially those related to public health spending), as well as of the estimation of such direct and indirect impacts; and (ii) the clustering of the Spanish autonomous communities according to their indicators for Well-being and the aforementioned constructs, so that the intra-country Well-being disparities due to regional public health spending policies can be visualized. This would allow us to estimate the legacy of the pandemic for the Spanish health system.

Finally, from a methodological point of view, another interesting research line is the development and estimation of the spatial and spatio-temporal versions of our PLS-SEM specification to consider the spatial or spatio-temporal dependencies existing in the healthcare and health spending databases. If such dependencies do in fact exist but are not accounted for, the estimates will not be accurate and their variances will be underestimated, which in turn implies an overestimation of the confidence levels and an underestimation of *p*-values, leading to incorrect rejections of the research hypothesis.

## Data availability statement

Publicly available datasets were analyzed in this study. This data can be found here: http://inclasns.msssi.es.

## Author contributions

MCVM: conceptualization and software. MCVM and J-MM: methodology and formal analysis. MCVM, MG, and J-MM: writing—original draft. MG: data curation. J-MM: writing—review and editing. All authors contributed to the article and approved the final manuscript.

## Funding

This work has been partially co-funded by the University of Almería, the University of Castilla-La Mancha and the European Regional Development Fund (ERDF): Grant PPUENTE2022/006 to the Research Group on Ethics, Gender and Sustainability (SEJ-647) and Grant 2021-GRIN-30983 (from European Union) to the Consolidated Research Group on Applied Economics and Quantitative Methods (ECOAP&MC).

## Conflict of interest

The authors declare that the research was conducted in the absence of any commercial or financial relationships that could be construed as a potential conflict of interest.

## Publisher's note

All claims expressed in this article are solely those of the authors and do not necessarily represent those of their affiliated organizations, or those of the publisher, the editors and the reviewers. Any product that may be evaluated in this article, or claim that may be made by its manufacturer, is not guaranteed or endorsed by the publisher.

## Footnotes

^1^In fact, not only the 2008 crisis. It is worth noting that the economic legacy left by President Zapatero enormously aggravated the effects of the crisis and was an important burden for the country. The enormous public debt and public deficit led the European Commission to force President Zapatero to cut welfare spending by 15 billion euros. Obviously, this poor economic legacy strongly conditioned the spending policy in the following years.

^2^The complexity of the model has little effect on the sample size requirements because the PLS-SEM algorithm does not compute all the relationships existing in the model at the same time but instead estimates partial ordinary least squares regressions.

^3^We use the terms “support” and “validation” in the sense of “no rejection”.

^4^Certain socio-economic factors are also popular in some research (GDP, economic inequalities, density of social and family networks…).
