# Supplementary material for: Regional well-being inequalities arising from healthcare expenditure public policies in Spain
Source: Front Public Health. 2022 Sep 21;10:953827. doi: 10.3389/fpubh.2022.953827 (PMC9533108; doi:10.3389/fpubh.2022.953827)
Supplement: Supplementary file 1 [file Data_Sheet_1.pdf]

Appendix 1. Mean of the variables by Autonomous Communities

Item	1	2	3	4	5	6	7	8	9	10	11	12	13	14	15	16	17
**WE1**	81.15	82.54	81.76	82.14	81.59	82.52	83.30	82.85	82.70	81.98	81.74	82.29	83.80	81.94	83.44	82.92	83.10
**WE2**	19.76	20.93	20.62	20.56	20.39	21.05	21.72	21.05	21.03	20.48	20.26	21.08	21.93	20.30	21.56	21.37	21.37
**MO1**	148.22	149.47	168.28	149.32	153.24	157.28	150.89	139.77	147.36	147.60	156.48	154.49	137.93	141.97	145.78	157.78	146.91
**MO2**	45.08	34.41	21.63	29.68	26.04	32.27	27.03	31.45	28.50	34.97	37.48	35.05	22.57	39.41	29.24	29.25	32.17
**MO3**	11.96	11.38	10.97	12.28	29.44	7.28	9.85	12.57	10.66	11.70	10.71	7.74	6.14	11.27	9.68	9.47	8.81
**EX1**	1204.49	1458.93	1509.15	1343.37	1359.92	1466.35	1431.43	1402.66	1340.03	1310.49	1514.97	1392.56	1238.01	1472.40	1520.92	1564.68	1479.54
**EX2**	58.43	58.77	61.91	62.49	60.35	58.75	54.89	54.63	59.82	57.72	53.61	58.22	66.44	60.25	59.73	60.15	58.74
**EX3**	3.76	2.68	3.55	2.58	2.93	4.51	3.46	3.08	2.73	4.01	2.72	3.29	4.86	2.89	2.56	2.98	3.22
**EX4**	20.17	18.57	18.98	14.99	18.16	17.41	19.07	19.52	17.37	22.08	20.05	22.92	16.34	18.59	16.14	16.28	16.85
**RE1**	1.39	2.00	2.00	1.62	1.40	1.68	1.72	1.74	1.81	1.55	1.67	1.66	1.77	1.62	1.79	1.81	1.67
**RE2**	0.74	0.86	0.75	0.64	0.72	0.78	1.07	0.83	0.73	0.72	0.87	0.81	0.69	0.72	0.77	0.72	0.82
**RE3**	1.85	3.01	2.92	2.16	2.12	2.32	2.67	2.29	3.01	2.13	3.07	2.89	2.11	2.07	2.30	2.66	2.80
**RE4**	5.88	6.85	7.20	5.67	4.57	6.34	6.33	5.53	6.92	6.71	7.60	7.30	6.37	6.32	6.84	6.83	6.66
**RE5**	0.24	0.22	0.34	0.24	0.18	0.40	0.23	0.26	0.48	0.24	0.23	0.27	0.29	0.23	0.29	0.35	0.22
**RE6**	1.05	1.34	1.29	0.91	0.76	1.16	1.03	1.30	1.07	1.11	1.55	1.30	0.97	1.13	1.21	1.17	1.04
**RE7**	0.39	0.59	0.71	0.57	0.29	0.59	0.53	0.53	0.63	0.57	0.68	0.68	0.74	0.51	0.41	0.45	0.77
**US1**	74.50	101.90	107.22	78.25	60.17	89.35	99.37	87.67	99.53	87.25	99.80	92.99	81.04	83.84	98.45	114.06	108.45
**US2**	7.19	7.44	7.95	6.88	8.56	7.53	7.44	7.25	6.40	6.08	6.53	8.35	7.61	6.89	6.89	6.86	6.28
**US3**	48.48	34.55	39.96	43.16	33.53	36.51	34.65	47.67	45.24	41.01	44.33	34.12	47.86	38.60	32.11	39.51	53.89
**US4**	65.44	74.83	66.72	58.60	41.83	55.82	72.42	65.24	93.51	75.99	76.50	71.58	72.78	59.49	71.92	87.98	79.51
**US5**	70.87	71.88	85.02	57.76	59.76	70.51	76.04	75.68	74.20	77.62	65.99	89.36	78.49	65.35	71.48	82.40	69.32
**US6**	25.60	22.91	39.57	26.94	16.21	30.27	26.95	32.19	31.03	41.25	26.63	34.38	42.38	17.36	22.14	15.99	39.85
**SA1**	4.74	4.53	5.05	3.55	4.57	4.97	4.97	4.67	3.56	4.07	4.59	5.18	4.07	3.74	4.09	4.19	3.70
**SA2**	1.89	1.82	1.77	1.52	1.79	1.88	1.66	1.81	1.48	1.62	1.63	1.91	1.59	1.80	1.18	1.52	1.19
**SA3**	228.93	818.76	871.71	491.80	375.75	694.64	251.06	184.29	299.77	261.82	336.25	360.22	460.83	236.95	911.61	548.12	334.34
**ED1**	18.15	24.88	21.22	24.27	20.30	21.38	21.70	18.85	26.76	21.20	16.39	20.06	30.62	20.14	28.16	30.16	25.39

1. Andalucía; 2. Aragón; 3. Asturias; 4. Baleares; 5. Canarias; 6. Cantabria; 7. Castilla y León; 8. Castilla-La Mancha; 9. Cataluña; 10. Comunidad Valenciana; 11. Extremadura; 12. Galicia; 13. Madrid; 14. Murcia; 15. Navarra; 16. País Vasco; 17. La Rioja

Appendix 2. Mean of the variables used as input for the AHCA

Item	Cluster 1	Cluster 2	Cluster3
**WE1**	82.88	83.62	85.43
**WE2**	21.03	21.65	23.14
**MO1**	134.71	141.39	118.33
**MO2**	25.83	23.53	15.95
**MO3**	10.72	8.35	4.72
**EX1**	1484.98	1612.71	1289.71
**EX2**	61.36	62.35	68.60
**EX3**	3.54	3.31	5.75
**EX4**	17.14	16.20	15.50
**RE1**	1.66	1.98	2.03
**RE2**	0.74	0.85	0.68
**RE3**	2.04	2.85	1.94
**RE4**	6.19	7.93	6.77
**RE5**	0.29	0.42	0.37
**RE6**	1.22	1.47	1.00
**RE7**	0.62	0.88	0.98
